# Early explorations of holistic review in graduate medical education

**DOI:** 10.5195/jmla.2025.2030

**Published:** 2025-01-14

**Authors:** Gena C. Dunivan, Jonathan D. Eldredge, Marlene P. Ballejos, Melissa Gonzales, Valerie Romero-Leggott

**Affiliations:** 1 gcdunivan@uabmc.edu, Professor of OBGYN, Director, Division of Urogynecology and Reconstructive Pelvic Surgery, University of Alabama at Birmingham, Birmingham, AL; When at UNM: Gena C Dunivan, MD, Associate Dean of Graduate Medical Education, Associate Professor of OBGYN, Urogynecology and Reconstructive Pelvic Surgery Fellowship Director, University of New Mexico, Albuquerque, NM; 2 jeldredge@salud.unm.edu, Professor, Health Sciences Library and Informatics Center and Family & Community Medicine, School of Medicine; Associate Program Director, Clinical Informatics, Graduate Medical Education, University of New Mexico, Albuquerque, NM; 3 mballejos@salud.unm.edu, Associate Professor in Family & Community Medicine and Assistant Dean for Admissions, University of New Mexico School of Medicine, Albuquerque, New Mexico; 4 Mgonzales1@tulane.edu, Professor and Chair. Department of Environmental Health Sciences, Celia Scott Weatherhead School of Public Health & Tropical Medicine, Tulane University, New Orleans, LA; When at UNM: Professor, Division of Epidemiology, Biostatistics and Preventive Medicine, Associate Director Preventive Medicine Residency Program, Associate Vice Chancellor for Research and Evaluation Office of Diversity Equity & Inclusion, University of New Mexico, Albuquerque, NM; 5 VRomero@salud.unm.edu, Vice President and Executive Diversity, Equity & Inclusion Officer; HSC Endowed Professorship for Equity in Health; Professor of Family & Community Medicine. Executive Director, UNM SOM Combined BA/MD Degree Program; UNM Health Sciences Center Office for Diversity, Equity & Inclusion

**Keywords:** Holistic review, Graduate medical education, Internship and residency, School admission criteria, Health care disparities, Cultural diversity, Population groups, Personnel selection, Social determinants of health, Social justice

## Abstract

**Background:**

Graduate Medical Education programs have implemented holistic review to improve the selection process for new residents. Holistic review will have a profound effect on Health Information Professionals (HIPs) with the arrival of medical residents with different backgrounds and needs. The unique experiences and skills of HIPs will position them well for the new realities in medical residency programs. This article traces the historic roots of holistic review.

**Methods:**

The authors employed a scoping review to track the historical traces of holistic review in Graduate Medical Education over the formative period of 1999–2019.

**Results:**

Medical residency programs over a 20-year period piloted holistic review in the screening, interview, and multiple time periods in the selection process. These ventures reflected a diversity of approaches and creative adaptations from other disciplines such as personnel management, organizational psychology, and active learning forms of education

**Conclusion:**

Health information professionals and medical educators will better engage with the newer cohorts of residents when equipped with a history of holistic review.

## BACKGROUND

The practice of holistic review has been implemented in medical education in recent years, particularly in Graduate Medical Education (GME). Holistic review already has had a major impact on selection processes for new medical residents. Holistic review will continue to affect the composition of medical residency programs and will profoundly affect how medical educators and Health Information Professionals (HIPs) interact with these residents. HIPs (health sciences librarians, informaticists, informationists, and archivists) have a long-standing history of working closely with GME programs in the US. Historically, HIPs have conducted literature searches to support patient care, clinical research, or bedside instruction such as patient rounds in support of GME programs. HIPs also have ensured that authoritative information resources are available for GME faculty residents, and fellows [1]. The more informationist and informatics-oriented HIPs have evaluated point-of-care resources and electronic health records. Some of these same colleagues administer GME Clinical Informatics Fellowships. HIPs' management skills have led to their involvement in the selection, evaluation, and education oversight GME committees at their institutions [2]. Some HIPs are sought out by GME programs for their curricular and instructional design skills. Over the past decade, HIPs have been closely connected with teaching medical residents and fellows in accordance with specialty-based ACGME Milestones [3]. HIPs also have demonstrated a commitment to diversity, equity, and inclusion for at least half a century [4–7]. These factors converge to make a deeper understanding of holistic review highly relevant to HIPs and their medical educator colleagues.

This scoping review traces the historical antecedents of current holistic review practices. It explains how these antecedents led to the quick and, perhaps surprising, rapid acceptance of holistic review.

The phrase “holistic review” in medical education refers to selecting candidates who will be well-matched to both the training program and to the program's patient populations. The Association of American Medical Colleges (AAMC) formally defines holistic review as a “flexible, mission-driven approach to recruit and assess an individual's competencies by considering their experiences, attributes, and metrics in order to select applicants who will best contribute to the program's unique goals, learning environment, and the practice of medicine [8].” The Accreditation Council for Graduate Medical Education (ACGME) in the United States (US) thoroughly embraced holistic review in late 2019 and encouraged Graduate Medical Education (GME) programs nationwide to institute holistic review in selecting applicants for specialty medical training. Societal concerns about health disparities fueled by health inequities disproportionately affecting women and minorities largely prompted and accelerated ACGME's commitment to holistic review [9].

Examining these early explorations of holistic review within GME programs holds significant historical and practical value to everyone connected to medical education in the US. In practical terms, the great enthusiasm for holistic review has not yet translated to many peer reviewed research articles on the subject so these early explorations have added pragmatic value. HIPs and clinical educators can learn a great deal about the early attempts to employ holistic review as they all probably have (or soon will be) been called upon to institute these practices given the groundswell in interest among GME programs. Finally, innovators in medical education can learn from how these, at the time, radical departures from standard selection practices contributed to a far-reaching reform movement in GME. In short, readers can gain valuable insights from this recent history.

Most readers associated with medical education recognize the profound influence of the residency Match process upon medical school curricula and medical students in the US. The Match was invented in 1951 to prevent medical students from exploitation by medical residencies. At that time, there were twice as many medical school graduates as there were available slots in residency programs, causing a severe power imbalance against the medical students. The Match consists of medical students submitting their lists of preferred ranked residency program choices while the medical residencies submit their preferred ranked choices of graduating medical students [10–12].

While this complementary ranking system stood the test of time, by the early 2000s the Match unintentionally led to mismatches of applicants with selected medical residencies. Part of the problem was the large number of residency programs to which individual medical students would apply. Faced with so many applicants, residency program faculty sought more efficient ways to streamline their screening processes. A quick yet unfortunate metric chosen to screen prospective residents turned out to be the United States Medical Licensure Exam (USMLE) Step 1. Residency programs in the process inadvertently succumbed to the quantitative fallacy by elevating an easily-measured score while ignoring other important factors [13–14]. The USMLE Step 1 has a mixed record in predicting students' success in residency programs [15]. Meanwhile, this one exam has caused unwarranted psychological stress for medical students [16]. As Salari and Deng note, “it is so symbolic that medical students fear the exam from the start of medical school.” They continue, “the infamous nature of Step 1 originates from the substantial weight scores carry in the resident selection process (p. 1,312).” rather than as an intended “checkpoint” of student progress in acquiring knowledge of the basic sciences [17]. In 2014, a total of 94% of medical residency program directors considered Step 1 to be a major factor in screening applicants. This overwhelming emphasis had the unintended consequence of suppressing needed medical school curricular change [18]. Importantly, the USMLE Step 1 perpetuated racial and gender disparities [19].

Diversity is of particular importance across all specialties as research supports that the quality of health care is improved when the providers' backgrounds or ethnicities reflect the community they serve [20–21]. Health care is far from this target: although minority groups represent 33% of the overall US population, the physician workforce is comprised of only 4.1% Black, 4.4% Hispanic, and 0.4% American Indian/Alaskan Native[22].

Fortunately, the USMLE Step 1 transitioned in 2022 to a pass/fail rather than scored exam. Holistic review emerged as a novel opportunity to align acceptances of medical school graduates with the needs of the residency programs and the needs of the patients served by these programs.

It will take a sustained, long-term effort to dismantle the structural and systemic sources that perpetuate subtle forms of racism in the US. The roots of racism can be traced back to the Doctrine of Discovery in the 1400s in Spain and Portugal then later adopted by other European nations and in the American colonies [23–25]. These structures and systems have taken centuries to accumulate and become entrenched [26]. Holistic review promises to erode these systems and to facilitate better matches between the needs of residency programs and their patients. In so doing, medical residencies will serve society's evolving needs [27].

### Research Question

What insights can be gained from early explorations into holistic review practices for selecting new residents in GME residency programs in the US prior to the broad endorsement of this approach in late 2019?

## METHODS

The authors selected the scoping review methodology to address the research question by providing a rapid, “preliminary map of the literature [28].” to understand the evolution of the revolutionary practice of holistic review. The authors referred to the Scoping Reviews protocol extensions to the Preferred Reporting Items for Systematic Reviews and Meta-Analyses (PRISMA-ScR) [29] and the Joanna Briggs Institute *Manual for Evidence Synthesis* throughout this scoping review [30].

### Data Sources and Searching

This scoping review presented challenges requiring creativity to retrieve the relevant early explorations of holistic review in GME. A search of PubMed on May 30, 2024 illustrates the challenges. The combined MeSH and adjacent textword search strategy of “Education, Medical, Graduate”[Majr] AND “holistic review” produced 68 references in PubMed. Only five references were published prior to 2020, although the concept of holistic review had existed for about two decades. The first use of the phrase “holistic review” among these five references that matched the formal concept first appeared in a 2016 article [31].

Three databases were searched to locate relevant studies due to their coverage of medical education references: Ebsco Cumulative Index to Nursing and Allied Health Literature (CINAHL), Ebsco Education Research Complete, and PubMed from the National Library of Medicine. The PRISMA flow diagram for scoping reviews in **Figure 1** and the detailed search strategies in the online **Appendix** provide many details.

All of the final searches in the three databases employed combinations of controlled vocabularies, keyword, and truncated keyword approaches. The first set of searches retrieved references intended to capture the concept of graduate medical education. The second set of searches identified references connected with either academic admissions or personnel selection since elements of both can occur within holistic review involving prospective residents. The final set of searches captured the range of attributes associated with holistic review. The three searches conducted during August 2020 were combined with AND to produce 635 references that were then filtered by the publication years 1999–2019 resulting in 513 references. The start date of 1999 was chosen because it was the first known attempt at holistic review and the end date of December 2019 was selected due to the endorsement of holistic review by ACGME [32]. This methodology text below and the **Appendix** will allow others to reproduce these searches in the future, per PRISMA guidelines and checklists for scoping reviews [33–34]. The Peer Review of Electronic Search Strategies (PRESS) checklist [35] proved helpful for replicability purposes even though it was originally intended for systematic reviews. The 513 references were uploaded into Rayyan, a web application for reviewing and critically appraising references. It should be noted for future searchers wishing to replicate this scoping review, that PubMed eclipsed the other two databases that either duplicated titles or contained articles to be excluded from final consideration. Supplementary tables in the online **Appendix** detail the initial reasons for rejecting the vast majority of the initially retrieved references.

### Inclusion and Exclusion Criteria

The first two authors (GD and JE) reviewed the abstracts of the 513 references in Rayyan with their choices concealed from one another. The authors rejected the majority of references (*n* = 444) mainly because they involved populations other than prospective medical residents (undergraduate medical education, dental, nursing, allied health, post-residency fellows, etc.), were from outside the US, or were preliminary works exploring the possibilities of holistic review; they also excluded editorials or commentaries. They agreed on all but 30 references when working in isolation with their choices concealed from one another. They reviewed these 30 references in Rayyan together in a meeting and resolved any differences. They agreed on 69 references to advance to the next stage. They attached PDFs of the actual articles to these 69 references for the next review phase.

Later, the authors removed 40 references on further examination of the articles themselves as these pertained more to background or rationales for holistic review.

## RESULTS

The remaining 19 selected articles within this scoping review reflect the wide range of diverse holistic review practices involving prospective medical residents during 1999–2019 in the US. **Table 1** outlines the historical progression of explorations with holistic review. The earliest instance of a holistic review was published by Thomas in 1999. It sought to correct for minorities and women historically underrepresented in medical residencies [36]. In the later periods, there were many more examples to consider.

**Table 1:** Chronology of Holistic Review Efforts

Year	First Author	Summary
1999	Thomas	Orthopedics department leadership insisted on greater gender and racial representation.
2009	Quintero	Diversified pool of orthopedic residents by selecting a range of Myers-Briggs personality types.
2010	Hemaida and Kalb	Decided on new residents based on non-cognitive factors and interpersonal skills identified during interviews.
2011	Bell et al.	Matched prospective residents' personalities to the surgery department's composite profile.
2015	Stephenson-Famy	Attributes of surgery residency interviews most likely to lead to successful residents.
2016	Schenker et al.	Validated a standard interviewing protocol that provided a more comprehensive picture of orthopedic surgery candidates.
2017	Bowe et al.	Family practice residency program directors identified traits of successful residents then used to create a ranking form to evaluate candidates.
2017	Martin and Salzberg	Delphi study to determine most desirable traits in family medicine residents.
2018	Schnapp	Emergency medicine candidates recorded a three-minute video designed to identify interpersonal skills and professionalism.
2019	McGuire	Structured interviews to identify desired traits and motivators for possible orthopedics residents.
2019	Shebrain	Compared cognitive with non-cognitive aspects of surgery residence candidates. Determined that cognitive traits were more important.
2019	Albana et al.	Increased diversity in internal medicine department by debiasing the interviewers and elevating desired non-academic traits in candidates.
2019	Butler et al.	Greater emphasis upon urology candidates' attributes and experiences and less emphasis upon academic performance.
2019	Garrick et al.	Eliminated USMLE Step One cutoff scores, increased diversity in interviewers, and recruited more diverse emergency medicine candidates.
2019	Spottswood et al.	Created comprehensive diversity recruitment plan that included a pipeline program, faculty diversity, and a standard selection process to recruit desirable radiology candidates.
2019	Wusu et al.	Structured interviews in family medicine residency did not include candidates' academic records and created a climate comfortable to candidates from underrepresented backgrounds.
2019	Byrd	Standardized video interviews to assess competencies in interpersonal communication and professionalism in emergency medicine candidates.
2019	Spector	Redesigned the interview process to remove potential bias in a neurology residency program.
2019	Villwock	Informatics techniques to evaluate emotional intelligence and personality profiles for a otolaryngology residency program.

Holistic review has been utilized at several time points: screening, the interview, and at multiple time points.

### Screening

The strategies utilized for the screening process focused on standardization of the process and options such as Standardized Video Interviews (SVI). Bird et al utilized the Association of American Medical Colleges (AAMC) SVI to help screen candidates for interviews by focusing on competencies such as interpersonal communication skills and professionalism, allowing the selection committee to differentiate candidates effectively [37]. Spector and Railey described their process to improve representation of under-represented in medicine (URiM) candidates, by implementing a process with no USMLE cut score. They used a point system for evaluating applicants for other characteristics based on other factors such as extracurricular activities/leadership, letters of recommendations, and life experiences. They reported the racial/ethnic discrepancies in interview offers decreased from 10.6% to 3.6% due these efforts [38]. Finally, Villwock et al. described the Selection Tool for Applicants to Residency (STAR) that uses a predetermined criteria algorithm to score different aspects of the applicants' Electronic Residency Application Service (ERAS) submissions to create an initial ranking for all applications. STAR weighs candidate characteristics on a 0 to10 point scale with weightings in parenthesis() : academic (1x), extracurricular activities (1x), research experience (3x), leadership positions (3x), and geographic connection (1x) to meet program needs. STAR offers a potentially more efficient selection process that avoids missing “diamonds in the rough” candidates who otherwise could be missed by focusing too intensely on USMLE step scores. Importantly, STAR did not disqualify eligible URiM and women candidates invited for an interview and there was no difference in resident attrition rates [39].

### Interview

Holistic review methods at the interview stage of selecting candidates for residencies rely on a range of approaches largely from the domains of personnel management and psychometric tests. Most of these holistic interviewing methods try to overcome interviewer subjectivity by providing structured formats. The interviewing methods described generally are future-oriented toward residents' actual specialty practice responsibilities rather than relying on past performance on standardized exams.

Bell et al. employed the TriMetrix Personal Talent Report (TPTR) to create an inventory of a surgery department's “behavioral styles, intrinsic motivators, and dimensional balance Page 534)” and a list of characteristics of superior performance. The characteristics in this profile that emerged pointed to the inadequacy of grades, exam scores, letters, and other traditional selection methods to predict success in residency (Pages 536 and 539) [40].

Bowe et al. created an Applicant Ranking Tool (ART) that aligned traits with the six ACGME competency areas along with essential non-cognitive areas gleaned from the interview including conscientiousness, curiosity, interpersonal skills, confidence, and recognition of one's limits. Additionally, creating an ART for one specialty does not necessarily mean it will translate to other specialties due to the different knowledge, skills, and values emphasized within varied specialties [41].

Hemaida and Kalb applied the Analytic Hierarchy Process (AHP), which offers an aid to making complex decisions, in selecting Family Practice residents. Family practice faculty, residents, and administrators who interacted with residents were asked to identify the most important six factors from a list of activities connected with selecting new residents. These factors were interview assessments, interpersonal skills, fit with current team, conformity with the organizational culture of the program, their personal statement, and alignment of future practice plans with program areas of emphasis. Respondents did not rank highly the prospective residents' medical school grades or USMLE scores [42]. Forman has provided an excellent background description of the AHP [43].

McGuire et al. structured prospective resident interviews according to critical tasks based upon a job analysis of successful orthopedic surgeons. The structured interviews also sought to determine the prospective residents' capabilities and motivations [44].

Quintero et al. used a prospective cohort study to identify bias in the selection of orthopedic surgeons by comparing the Myers-Briggs personality types of interviewers and interviewees. This can be employed to avoid too much convergence of personality types within a program [45]. Past studies have noted the clustering of certain personality profiles within any given medical specialty [46–48]. Schnapp et al. used a Standardized Video Interview (SVI) to evaluate 125 prospective emergency medicine residents' interpersonal and professionalism skills. Candidates video recorded their three-minute responses to a series of six standard questions posed by program faculty members. These scores were compared with faculty gestalt scores based upon in-person unstructured faculty interviews. There was no significant correlation between the SVI scores and the faculty gestalt scores, suggesting that the two formats are measuring different aspects of professionalism and interpersonal skills [49].

Shebrain et al. discovered that USMLE Steps 1 and 2 scores had an inverse relationship in their program for predicting candidates' success during in-person interviews. Non-cognitive aspects, particularly those presented during the interview were far more predictive of resident candidate success in securing favorable rankings in the interview scores. The non-cognitive aspects consisted of letters of recommendation, personal statements, how the candidate represented oneself, the candidate's stated interest in the specific program, responses to standardized questions, and the degree of connection an interviewer felt with the interviewee. This latter connection proved to be the only predictive factor when controlling for all other variables [50].

Stephenson-Famy et al. determined the importance of the interview in the selection process based on a literature review of 104 studies. The interview assesses non-cognitive attributes such as communications skills, maturity, and professionalism. The authors recommended that a rigorous and structured interview strategy should replace the unblinded and unstructured interviews of the past. The structured interview should include: a written description of desired traits, standardized questions, behavior-specific anchors with a scoring rubric, multiple observers, interview trainings to avoid unethical questions, and blinding of the interview to academic metrics [51].

Johns Hopkins Department of Orthopedic Surgery committed to a holistic review of prospective candidates that emphasized candidates' potential to succeed rather than their national exam score metrics. Candidates' diverse backgrounds, interpersonal skills, openness to new learning, and work ethic instead were the focus of onsite interviews. African-American and women residents scored no differently than other residents in multiple assessment events and the board certification exams [52].

### Multiple Time Points

Many of the studies that utilized a holistic process included similar key features in a multipronged approach at different times during the application process, including recruitment, screening, interviewing, and ranking. Outreach to the desired applicant pools (For example, URiM medical students) was mentioned by several articles [53–54]. Having standardized “themes” to discuss in interviews was discussed by Schenker et al. Each interviewer was assigned a theme including knowledge, affective domain, ethics, research, and “fit” [55]. Most articles also emphasized the importance of standardized screening tools that decreased emphasis on traditional academic metrics coupled with structured interviews [56]. A unique component discussed in these articles was that the representation of URiM residents and faculty in the process was critical. This could include being present at recruitment events, interviews or sending a personal email or follow-up phone call [57]. Garrick et al. highlighted an annual diversity recruitment dinner in which URiM applicants in emergency medicine, internal medicine, and surgery could attend to demonstrate the hospital's support for diverse residents [58]. Also discussed was sponsoring a no cost “second-look weekend” for highly desired applicants [59]. These articles exemplify a multi-pronged approach to holistic review while working to increase diversity in a program. An example highlighting this success can be seen in Garrick et al. Not only did their multi-pronged approach increase the proportion of URM graduating from their residency (12% to 27%) over an eleven-year period, but the authors note that during that time all residents graduated on time and the program has a first time pass rate of 98%.

## LIMITATIONS

There are a number of limitations to this study. First, authors of these documented studies varied in their operational definitions of “holistic review” practices. These authors generally referred to practices intended to review prospective medical residents beyond primarily exam scores or other systems rigidly adhering to quantitative scores or comparative ranking as holistic review. Second, this scoping review included only studies conducted prior to the Covid-19 pandemic, thereby excluding all of the subsequent changes in medical residency recruitment, screening, and selection practices. Third, the identified study designs and approaches of holistic review varied widely, posing challenges for any close comparisons. In addition, the limited numbers of studies make it difficult to draw meaningful outcomes.

In addition, when programs are seeking to improve the number of URiM candidates that apply and ultimately match with their programs, other tools identified to increase success, included visiting clerkships, directed outreach to national organizations, voluntary presence of faculty and/or residents that identify as URiM at interviews with personal follow-up, and expense paid second look visits.

Despite the lack of standardization in how residency programs approach the concept of holistic review, it is clear that new processes are needed, especially now that the USMLE Step 1 has transition to pass/fail. The AAMC provides tools and resources for programs to begin crafting their unique approach to holistic review. It will be critical to follow outcomes to determine if the goals of holistic review have been achieved, including training residents who will thrive in their particular specialty, reflect the communities they serve, and broaden diversity across training programs to improve health access and outcomes for diverse populations.

## CONCLUSION

Holistic review should be implemented throughout the recruitment, screening, interviewing, and ranking phases. Utilizing it at only one time point may still result in bias and hinder some candidates from moving to the next stage of selection. HIPs often serve on these selection committees and can offer their own social justice perspectives. Holistic review offers concrete ways to further diversify their ranks through recruitment and hiring efforts.

The main themes and recommendations include training and buy-in from faculty and other stakeholders involved in the recruitment efforts, development of qualities desired from candidates, attributes felt to be associated with success in the field, mission alignment, and priorities for individual programs such as commitment to an underserved area or bilingual proficiency. Standardized screening based on a predetermined scoring system with a decreased focus on academic metrics, coupled with structured interviews that are masked to academic metrics should be considered along with a decision aid or ranking tool that allows differential weights to be applied to particular capabilities that the program deems as “highly important”. Finally, it is critical to follow outcomes to determine if the goals of holistic review have been achieved, including training residents that will thrive in their particular specialty, reflect the communities they serve, and broaden diversity across training programs to improve health access and outcomes for diverse populations.

Given the variability in the literature, it is somewhat difficult to offer an integrated synopsis. Key take-aways are that the majority of the 1999–2019 literature focuses on “fit” into residency and attempting to predict who will be successful in that residency. Performing holistic review during the screening process mainly highlighted ways that programs have decreased reliance on traditional academic metrics which did result in increased diversity in the applicants offered interviews. The main goal of utilizing a holistic process during the interview phase was to implement strategies to reduce interviewer subjectivity and unconscious bias. These strategies included standardized questions, bias training, and a variety of ranking tools. Holistic review involving multiple time points typically utilized standardized tools. All of these studies point to the need for longer term follow-up to both assess if utilization of holistic review tools resulted in more inclusive applicants and residents, but particularly if holistic review helps identify people who will ultimately be successful in that field. With their pragmatic sensibilities and their long-standing history of a commitment to equity and inclusion, HIPs can immediately grasp the benefits of and pivot to collaborate with the new residents selected due to holistic review.
